# Association of respiratory failure with inhibition of NaV1.6 in the phrenic nerve

**DOI:** 10.1080/19336950.2022.2122309

**Published:** 2022-10-14

**Authors:** Rebecca M. Klein, Mark E. Layton, Hillary Regan, Christopher P. Regan, Yuxing Li, Tracey Filzen, Matt Cato, Michelle K. Clements, Jixin Wang, Raul Sanoja, Thomas J. Greshock, Anthony J. Roecker, Joseph E. Pero, Ron Kim, Christopher Burgey, Christopher T. John, Ying-Hong Wang, Neetesh Bhandari, Arie Struyk, Richard L. Kraus, Darrell A. Henze, Andrea K. Houghton

**Affiliations:** Merck Research Laboratories, Merck & Co., Rahway, NJ, USA

**Keywords:** Sodium channel inhibitor, respiration, selectivity, phrenic nerve

## Abstract

As part of a drug discovery effort to identify potent inhibitors of NaV1.7 for the treatment of pain, we observed that inhibitors produced unexpected cardiovascular and respiratory effects in vivo. Specifically, inhibitors administered to rodents produced changes in cardiovascular parameters and respiratory cessation. We sought to determine the mechanism of the in vivo adverse effects by studying the selectivity of the compounds on NaV1.5, NaV1.4, and NaV1.6 in in vitro and ex vivo assays. Inhibitors lacking sufficient NaV1.7 selectivity over NaV1.6 were associated with respiratory cessation after in vivo administration to rodents. Effects on respiratory rate in rats were consistent with effects in an ex vivo hemisected rat diaphragm model and in vitro NaV1.6 potency. Furthermore, direct blockade of the phrenic nerve signaling was observed at exposures known to cause respiratory cessation in rats. Collectively, these results support a significant role for NaV1.6 in phrenic nerve signaling and respiratory function.

## Introduction

Voltage-gated sodium channels (NaV) form a large family of proteins, of which there are nine known NaV subtypes, NaV1.1 – NaV1.9, with widespread expression throughout the body. The primary functions of NaVs are thought to be initiation and propagation of action potentials in neuronal dendrites and axons, respectively [1]. Each of the NaV subtypes are differentially expressed throughout the body and are known to have different pharmacological roles in the nervous system. For example, NaV1.1 and NaV1.2 are broadly expressed within the central nervous system, while NaV1.4 and NaV1.5 are restricted to the periphery in muscle tissue and the cardiomyocytes of the heart, respectively.

The remaining subtypes, NaV1.6–1.9 are expressed in overlapping populations of peripheral neurons from the dorsal root ganglia (DRG). In addition to mechanosensitive neurons, NaV1.6 has also been detected in the phrenic nerve, motor neurons, and the CNS (nodes of Ranvier) [2,3]. NaV1.7 is present in the majority of DRG neurons, [4] and also expressed in sympathetic and olfactory sensory neurons, and is the main sodium channel underlying olfactory transmission to the bulb [5,6]. The neuronal expression and contribution to neuronal signaling of each of these peripheral subtypes in nociceptors has supported a role for each in pain sensation. NaV1.6 exhibits a special property termed a resurgent current, that has been implicated in nociceptor hyperexcitability in pain states [7,8]. NaV1.7 and NaV1.9 are thought to set the threshold for action potential firing in nociceptors, while NaV1.8 is thought to be responsible for action potential propagation.

Sodium channel inhibition is widely regarded as a validated approach to treating pain based upon a large body of human pharmacological and genetic evidence. Nonselective sodium channel inhibitors are routinely used in clinical practice as short acting local anesthetics. Naturally derived toxins, like tetrodotoxin (TTX) and ProTx-II, have also demonstrated pre-clinical analgesic efficacy and clinical sensory deficits [9,10]. The discovery of human genetic mutations that result in extreme pain phenotypes further validates the use of voltage-gated sodium channels inhibitors for the treatment of pain. Loss-of-function mutations in NaV1.7 and NaV1.9 have been demonstrated to cause congenital insensitivity to pain (CIP) disorders where affected patients have an inability to feel any pain [11–14]. Gain of function mutations in NaV1.7 result in either inherited erythromelalgia (IEM) or paroxysomal episodic pain disorder (PEPD), while NaV1.8 mutations are found in a small population of idiopathic small fiber painful neuropathies [15,16].

Clinical experience with sodium channel inhibition points toward the need for subtype selectivity. Local anesthetics and other nonselective sodium channel inhibitors like carbamazepine and lacosamide, are described as being “dose-limited” [17]. Their efficacy is tempered by activity on some NaV subtypes that limit the dose that can be administered. Despite its analgesic potential, TTX ingestion in humans can be poisonous and can lead to paralysis and respiratory failure if not treated immediately [9,18]. The serious systemic effects of TTX on muscle and respiratory function have been linked to its inhibition of sodium channels [10], likely NaV1.4. The effects on the central nervous system by nonselective sodium channel inhibitors and the expression patterns of the NaV subtypes has led to the hypothesis that selective sodium channel inhibitors – targeting only those subtypes expressed in peripheral nociceptors – might maintain the analgesic effects of nonselective inhibitors while achieving a much greater therapeutic window.

Given the need for novel analgesics and the increasing body of evidence that selective Nav1.7 channel inhibition would afford profound analgesia, we sought to identify novel selective Nav1.7 channel inhibitors [19,20]. Any leads needed to be highly selective over both NaV1.5 and NaV1.4 to limit cardiac adverse effects specifically prolonged PR and QRS intervals and to limit impact on muscle function, respectively. To limit CNS toxicity, compounds were optimized to have selectivity over NaV1.1 and NaV1.2 channels and/or physicochemical properties that limited central penetrance or exposure. At the outset, there was limited literature suggesting that NaV1.6 represented a liability, therefore selectivity over NaV1.6 was not initially determined as a requirement for inhibitors. We hypothesized that NaV1.6 inhibition was a property that might confer added efficacy to a NaV1.7 inhibitor, consistent with its expression in peripheral nociceptors. Contrary to our expectations, as potent NaV1.7 compounds were synthesized with varying degrees of NaV1.6 potency, we observed cardiorespiratory effects that appeared to be correlated with Nav1.6 potency. These data highlight the need for achieving selectivity over Nav1.6 for future drug-discovery efforts in sodium channel inhibition.

## Materials and methods

### Compound preparation

**Figure 1.**
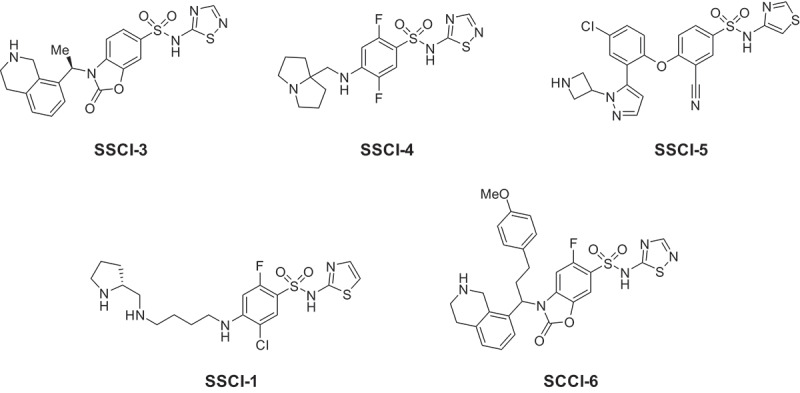
Structures of NaV1.7 inhibitors.

The structures of the compounds studied in this report are shown in Figure 1. Synthesis of **SSCI-3** [19], **SSCI-4** [20], **SSCI-5** [21], **SSCI-1** [22] have been previously reported, and synthesis of **SSCI-6** is reported in the SI. For in vitro electrophysiology, compounds were prepared from 10 mM stock dimethyl sulfoxide (DMSO) solutions. Compounds were pipetted onto a 96-well plate, and external solution added to achieve the final concentration in 300 uL total volume. The final DMSO concentration in external solution did not exceed 0.3%.

For rat hemisected diaphragm (HMD) experiments, compounds were prepared from 30 mM stock DMSO solutions. Compounds were diluted to their final concentration in external solution, then applied to the recording chamber. The final DMSO concentration did not exceed 0.1%.

For in vivo experiments (rat cardiovascular/respiratory and phrenic nerve), compounds were prepared in 100% dimethyl formamide (DMF) and administered via intravenous infusion.

### NaV1.x electrophysiology

Sodium currents were recorded from recombinant Human Embryonic Kidney 293 cell lines stably overexpressing the respective sodium channel type (NaV1.2, 1.5, 1.4, 1.6, 1.7). Recording solutions were comprised of the following (in mM): Internal solution: 30 CsCl, 5 HEPES, 10 EGTA, 120 CsF, 5 NaF, 2 MgCl_2_, pH = 7.3 with CsOH; External solution 1: 40 NaCl, 120 NMDG, 1 KCl, 0.5 MgCl_2_, 5 HEPES, 2.7 CaCl_2_, pH to 7.3 with NaOH, used for NaV1.5 recordings; External solution 2: 150 NaCl, 5 KCl, 2 CaCl_2_, 1 MgCl_2_, 10 HEPES, 12 Dextrose, pH 7.3 with NaOH, used for NaV1.7, 1.6, 1.4, and 1.2 recordings.

Whole-cell currents were recorded from cells stably expressing NaV channels using the PatchXpress 7000A automated patch clamp platform (Molecular Devices, LLC; Sunnyvale, CA). A portable air-conditioning unit was installed on the PatchXpress instruments to maintain the temperature of the headstage at 21°C. Cells were voltage clamped at −60 mV for cell detection and sealing. At the start of each recording, a voltage curve and an inactivation curve was run for each cell to determine the voltage at which 50% of the channels reside in the inactivated state (V_0.5 inact_). The voltage curve was used to determine if the cell was adequately clamped; cells with currents larger than 10nA or with space clamp issues were not used further. Compound potency was determined using different voltage-clamp protocols as described below for each channel subtype.

*Na_V_1.7, Na_V_1.6, Na_V_1.4*: Compound potency was measured in a hyperpolarized-state protocol where the holding potential for each cell was set to V_0.5inact –_ 20 mV. A pulse train consisting of 7 consecutive double pulses, 8 second hypepolarizing prepulse to −120 mV followed by a test pulse to −10 mV was applied at a frequency of 0.1 Hz. First, vehicle (0.3% DMSO) was added to establish a baseline measurement. Once baseline was established, test compounds were added. Cells were exposed to compound for 5 minutes at 20 mV negative to V_0.5inact_ then exposed to the same hyperpolarized voltage protocol. (no pulsing during compound incubation). A washout was performed to measure recovery of the sodium currents from inhibition.

*Na_V_1.5*: Compound potency was measured in a depolarized-state protocol where the holding potential for each cell was set to V_0.5inact –_ 20 mV. The pulse protocol consisted of an 8 sec prepulse to V_0.5inact_ + 7 mV, followed by a 2 ms hyperpolarization to −120 mV and a 10 ms test pulse to −10 mV. First, vehicle (0.3% DMSO) was added to establish a baseline measurement. Once baseline was established, a test compound was added. Cells were exposed to compound for 5 minutes at V_0.5inact –_ 20 mV. Compound washout was performed to measure the recovery of the sodium currents from inhibition.

*Na_V_1.2*: Cells were held at a potential of V_0.5inact –_ 20 mV and depolarized to −10 mV at a frequency of 2 Hz. First, vehicle (0.3% DMSO) was added to establish a baseline measurement. Once a baseline was established, a test compound was added. Cells were exposed to compound for 5 minutes at V_0.5inact –_ 20 mV. A washout was performed to measure the recovery of the sodium currents from inhibition.

Recordings were included for analysis when current amplitude reached steady-state in vehicle, compound inhibition was concentration-dependent and currents recovered during wash-out periods. Raw data from PatchXpress experiments were retrieved from the database and analyzed using the DataXpress 2 software (Molecular Devices). Recordings were rejected when V_0.5inact_ was out of range (V_0.5inact_ NaV1.7: −77 mV ± 20 mV, V_0.5inact_ NaV1.6: −77 mV ± 20 mV, V_0.5inact_ NaV1.5: −89 mV ± 20 mV, V_0.5inact_ NaV1.2: −55 mV ± 20 mV; determined by historical reference data for each subtype), vehicle or test compound recordings did not reach steady-state after repeated pulsing due to current run-down or run-up, or test compounds did not show dose dependence of inhibition. Accepted recordings showed peak current amplitudes in vehicle ≥ 0.3 nA, membrane resistance, Rm >300 MOhm and access resistance, Ra < 20 MOhm .

Percent inhibition at each compound concentration was determined using the following equation: % inhibition of I_Nav_ = 100*(I_Nav(control)_-I_Nav(drug)_)/I_Nav(control)_; where I_Nav_ peak current amplitude measured at the test pulse during sweep 1. At least 3 concentrations (3–9 replicates per concentration) were tested and at least one concentration had to cause 20–80% current inhibition. Furthermore, the standard error for measurement at each concentration had to be below 10%. IC_50_ values were calculated using a four parameter logistic function (Hill equation): f(x) = I_min_ + (I_max_-I_min_)/(1+(IC_50_/[x])^h^); where IC_50_ = half maximal inhibitory concentration; I_min_ = minimal current (fixed to 0), I_max_ = maximal current (fixed to 100), h = Hill coefficient (fixed to 1).

### Animal use and care

All aspects of the work including housing, experimentation, and animal disposal were performed in general accordance with the “Guide for the Care and Use of Laboratory Animals: Eighth Edition” (The National Academies Press, Washington, DC, 2011) in our AAALAC-accredited laboratory animal facilities. All animal protocols were approved by the research laboratories of Merck & Co., Inc., Rahway, NJ, USA (MRL) IACUC committee, whether they were performed within MRL or at partner contract research organizations.

### Rat hemisected diaphragm assay

Wistar derived male or female rats weighing 450 ± 50 g were euthanized by CO_2_ overexposure. The diaphragm with the phrenic nerve attached was removed and placed into a tissue recording chamber containing Krebs solution pH 7.4; (composition in g/L: NaCl 6.89, KCl 0.35, CaCl_2_ 0.277, KH_2_PO_4_ 0.163, MgSO_4_ · 7H_2_O 0.296, NaHCO_3_ 2.1 and glucose 1.8). A piece of cotton was attached to the top of the phrenic nerve over the jaw electrodes. A second piece of cotton was attached to the tip of the tendinous tissue at the apex of the diaphragm preparation for attachment to an isometric transducer under 1 g tension. The rib of the diaphragm preparation was secured to the bottom of the jaw electrodes. The Krebs solution in the recording chamber was bubbled with 95% O_2_, 5% CO_2_ at a rate which adequately aerated the solution but did not cause violent turbulence of solution and movement of the diaphragm. The recording chamber was placed into a water bath kept at a temperature of 32°C by means of a suitable thermoregulator consisting of a thermostat and heating elements in addition to a water circulator.

The isometric transducer was connected to an amplifier and the tissue response was recorded on a two-pen recorder set at 1 volt. Field stimulation [S48 Stimulator (GRASS) set at 0.2 Hz (12 pulses per minute), 0.5 ms pulse duration at 70% of maximum voltage (about 3 to 5 volts)] delivered to two circle platinum electrodes (0.2 cm in diameter) was applied for transmural tissue stimulation. Each tissue preparation was washed three times in Krebs solution every 15 minutes for a 60 minute equilibration period and permitted to develop contractions. D-Tubocurarine (300 µM dissolved in 0.1 mL distilled water for a final 3 µM bath concentration) was added to establish an isometrically recorded control relaxation response ranging from one to two centimeters and corresponding to 60–70% of maximal relaxation. In order to normalize across preps, we operationally defined the magnitude of the relaxation seen with turboC to be 100% inhibition. The tissue was then washed periodically until the contraction returns to the baseline value.

Sixty minutes later, the test compound was added (e.g. 30 mM in 0.01 mL vehicle of 100% DMSO, for final concentration of 30 µM test compound and 0.1% DMSO). A test compound recorded relaxation response of 50% or more (≥50%) within 5 minutes, relative to the 3 µM d-Tubocurarine control response, indicates neuromuscular blocking activity.

These studies were carried out by Eurofins Panlabs Taiwan, Ltd. on behalf of Merck & Co., Inc., Kenilworth, NJ, USA and in accordance with an approved Animal Procedure Statement from the MRL IACUC.

### Rat Cardiovascular/Respiratory (CV/R) assay

Male CD Rats were anesthetized with ketamine:xylazine (85 mg/kg:5 mg/kg, im, to effect). The left femoral artery and vein were cannulated for the measurement of blood pressure and the administration of test agent, respectively. The right femoral vein was cannulated for maintenance anesthetic infusion (ketamine:xylazine ~35 mg/kg/hr:1.5 mg/kg/hr, iv, to effect). Needle electrodes were placed subcutaneously at the right axillary and left inguinal areas to record lead II ECGs. When ECG intervals were required to determine effect on cardiac conduction a pacing/recording electrode catheter was advanced through the right jugular vein to pace the heart. ECG intervals were then measured at a fixed cardiac rate of 400 beats/minute to remove any variability of intrinsic heart rate on these parameters.

To determine the test agent-dependent effect on respiratory rate, a tracheotomy was also performed using a short length of polyethylene tubing containing a thermistor (AD Instruments). The change in temperature detected by the thermistor was used to calculate respiratory rate.

### Rat phrenic nerve firing assay

To determine direct effects on phrenic nerve activity, male CD rats were anesthetized and instrumented as described above for the CV/R assay. In addition, an incision was made in the ventral neck area and the right phrenic nerve identified anatomically and isolated via blunt dissection. The phrenic nerve was placed over a high impedance bipolar hook electrode and the nerve covered with mineral oil to prevent desiccation. Animals were mechanically ventilated via a tracheotomy (~10 mL/kg, 40–70 breaths/min, titrated individually to stabilize phrenic activity) following neuromuscular block (pancuronium 0.5–1 mg/kg, iv) and bilateral vagotomy to better define test agent effects on phrenic nerve activity. Anesthesia was verified repeatedly following pancuronium administration by testing the BP/HR response to noxious stimuli. Spontaneous electrical activity was continuously recorded and then later rectified and averaged to calculate frequency, width, peak height of bursts, and magnitude. Magnitude of the activity was calculated by taking the absolute value of the raw signal and then averaging that signal over 50 ms increments. The resulting average signal was then converted to magnitude by subtracting the maximum signal from the minimum signal for each phrenic burst complex. The resulting magnitude values were then averaged over 1 minute increments to result in the graphs shown. Figure S1 in the Supplemental Information illustrates this analytical procedure.

For in vivo CVR and phrenic nerve activity studies, all raw signals were amplified via general purposed biological amplifier (filter settings DC – 3-5 KhZ) then digitized, calibrated and collected at a minimum of 500 Hz (1000 Hz for ECG and 2000 Hz for cardiac electrograms, and 10 KHz for phrenic nerve activity) using Notocord-HEM data acquisition and analysis software. The raw signal was filtered following acquisition with a bandpass filter set to 200–800 Hz). Vehicle (100% dimethylformamdide, DMF) or compound formulated in the vehicle was administered as a 30-minute IV infusion at 0.0167 μL/g body weight/min. Doses were selected to achieve unbound plasma concentrations in range of measured Nav1.6 IC_50_ values when infused at 0.0167uL/g body weight/min based on measured rat clearance, plasma protein binding and Nav1.6 IC_50_. Exposure in rats was determined in separate pharmacokinetics experiments where compounds were administered via IV dosing.

## Results

### NaV1.x inhibition by small molecules in electrophysiology demonstrate a variety of selectivity profiles

**Figure 2.**
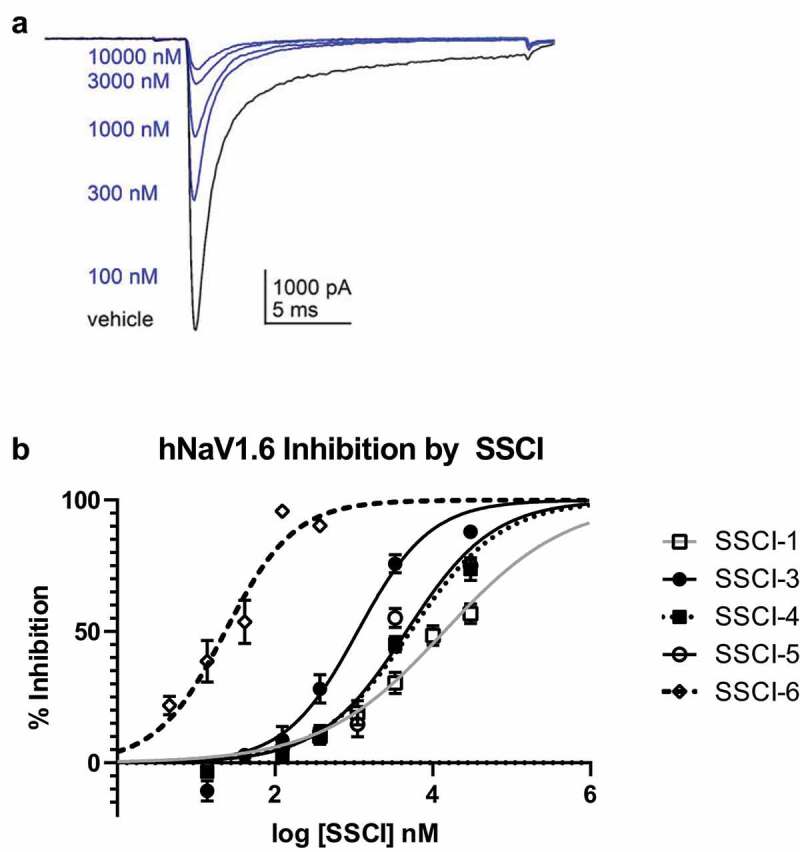
**Inhibition of NaV1.6 currents in HEK-293 cells**. (a). Representative manual electrophysiology traces for **SSCI-4** applied to HEK-293 cells stably expressing human NaV1.6 as described in the Methods. Five concentrations of **SSCI-4** are shown (100, 300, 1000, 3000, and 30,000 nM, blue traces) and a vehicle trace (black trace) obtained prior to compound application. (b). In vitro potencies were measured as described in the Methods. For each SSCI, then percent inhibition is plotted as a function of log concentration, then fit with the Hill Equation to determine the IC_50_ value for that compound.

As part of the screening process to identify novel inhibitors of NaV1.7, compounds were tested against several other voltage-gated sodium channels. Inhibition was assessed on both human and rat channels overexpressed in HEK cells using the PX automated electrophysiology platform. All compound potencies were measured in a hyperpolarized protocol as described in the methods. Prior experiments had determined that the hyperpolarized potency of compounds on NaV1.7 was a good predictor of their in vivo analgesic efficacy across several different preclinical animal models – the in vitro IC_50_ was predictive of the in vivo IC_90_ [23]. The calculated IC_50_ values for five compounds (**SSCI-1**, **3**–**6, Figure 1**) are reported in Table 1. As shown in Figure 2b, concentration-response curves for inhibition of human NaV1.6 were generated for **SSCI-1, 3–6**. Representative electrophysiology recordings for compound **SSCI-4** are shown in Figure 2a. All five compounds were additionally screened against hNaV1.5 for which they were inactive at concentrations up to and including 30 μM. **SSCI-1, 3–5** were inactive against hNaV1.4 up to 30 μM and demonstrated a high degree of selectivity against a broad panel of different ion channels (see Supplemental Information Table 1). In addition, compounds from this class of aryl-sulfonamides are known to have peripheral restriction due to poor passive permeability and active transport by Pgp. **SSCI-1, 3–5** displayed such low passive diffusion that Pgp transport could not be accurately assessed. Earlier work on **SSCI-3** and related compounds reported the peripheral restriction of these compounds [21]. **SSCI-1, 4–6** also have confirmed that central exposure, measured through CSF concentrations in mouse, was low for this class of compounds [20,23].

**Table 1.** Human NaV1.x potency for SSCIs.

Compound	Human NaV1.7 IC_50_ (μM)	Human NaV1.6 IC_50_ (μM)	Rat NaV1.6 IC_50_ (μM)	Human NaV1.5 IC_50_ (μM)	Human NaV1.4 IC_50_ (μM)
SSCI-3	1.0	1.113	8.1	>30	>30
SSCI-4	5.0	5.216	23	>30	>30
SSCI-5	2.1	4.614	7.4	>30	>30
SSCI-1	0.065	>30	19	>30	>30
SSCI-6	0.29	0.023	>0.041^a^	>30	ND

^a^45% inhibition at 41 nM

### Cardiac and respiratory effects observed in rodent studies

**Figure 3.**
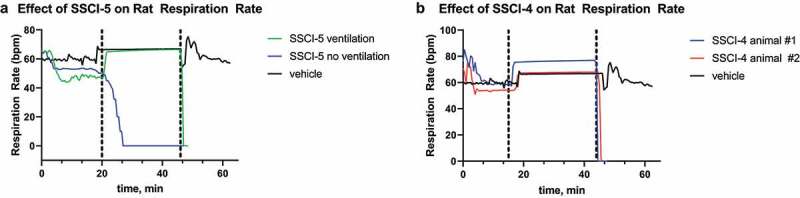
Respiratory effects of **SSCI-4** and **SSCI-5** in anesthetized rats.

To investigate the selectivity to cardiovascular effects, the compounds were profiled in the rat CV/R assay as shown in Table 2 for all compounds. In this assay respiratory rate, heart rate, blood pressure and ECG following cardiac pacing were measured in anesthetized rats during IV administration of compound. **SSCI-3** surprisingly showed dose-dependent mortality after 20–21 minutes when administered at 30 mg/kg, resulting in unbound plasma exposure of 20.2 μM just prior to death. No mortality was observed when **SSCI-3** was administered at 10 mg/kg. We then tested the structurally diverse compound **SSCI-5** in the rat CV/R assay and observed similar effects on the parameters measured at similar exposures resulting in mortality. The unbound plasma concentrations measured from blood samples taken just prior to death was 19.7 μM for **SSCI-5**. Preliminary studies with **SSCI-4**, another structurally diverse compound, exhibited similar results to **SSCI-5** (Table 2, unbound plasma concentration of 18.7 μM). In contrast, **SSCI-1** at similar unbound plasma exposures (16.9 μM) had minimal effects on cardiovascular and respiratory parameters. No mortality was observed with **SSCI-1**. Of the compounds tested, **SSCI-6** resulted in mortality at much lower unbound plasma concentrations compared to **SSCI-3–5** (**SSCI-6** unbound concentration of 0.05 μM measured from blood samples collected just prior to death). In the rat CV/R assay, **SSCI-6** decreased the respiratory rate by approximately −17 to −18% at unbound plasma exposures close to its measured NaV1.6 IC_50_ (Table 3). At exposures less than 2-fold higher, **SSCI-6** produced complete respiratory cessation and mortality. Generally, the in vivo profile of these compounds following IV administration included modest changes in respiratory rate and hemodynamics up to an exposure threshold at which point marked cardiorespiratory collapse was observed. For example, **SSCI-5** infusion resulted in mild respiratory depression with mild to no effect on arterial blood pressure and heart rate initially. However, as exposures increased, marked and rapid changes in both cardiac and respiratory parameters precluded both accurate determination of changes and identification of the primary physiologic mechanism. We then conducted an observational study with **SSCI-5** infusion ± ventilation to understand if mortality was driven either by primary cardiac or respiratory effects. As shown in Figure 3a, ventilation was initiated after initial respiratory decreases in a **SSCI-5** administered animal (green) and at a similar time post-infusion started in a vehicle-treated animal (black). Without ventilatory support, respiratory (Figure 3a, blue) and cardiac (not shown) collapse was observed within 30 min. However, ventilatory support enabled administration of higher doses of **SSCI-5** without effects on hemodynamics or QRS interval (data not shown) but animals were unable to regain spontaneous respiratory activity in contrast to vehicle-infused animal (Figure 3a). These data strongly pointed to the respiratory system, either through nerve conduction or neuromuscular junction effects, was the main physiologic mechanism of unexpected mortality. Similarly, animals infused with **SSCI-4** (Figure 3b), showed a respiratory collapse when ventilator support was withdrawn (red and blue traces).

For **SSCI-5** (A) and **SSCI-4** (B), respiration rate (breaths per minute, bpm) is plotted versus time (minutes, min) for the in vivo rat CVR assay. In both panels, the black traces represent the respiration rate in animals that were only treated with vehicle (DMF) over 60 minutes. The dashed vertical lines represent the start and end of supplemental ventilation for animals in the black and green trace (A) or red and blue traces (B). In A, the blue trace is the respiration rate during infusion with **SSCI-5** at 30 mg/kg without ventilation. The green trace is the respiration rate during infusion with **SSCI-5** at 30 mg/kg with ventilation. In B, the blue and red traces are the respiration rate in two animals during infusion with **SSCI-4** at 10 mg/kg with ventilation. The black trace represents a vehicle (DMF) treated animal. Blood samples were collected for test agent exposures at selected times in the study (see Results). For all traces, infusion of either vehicle or compound was started at 0 minutes; a stable baseline in vehicle was obtained before the start of the infusion and is not represented here.

**Table 2.** In vivo rat respiratory profiles of **SSCI-1, 3–6** following IV infusion.

Compound	Dose (mg/kg)	Time (min)	n	Total [plasma] (μM)	Unbound [plasma] (μM)	∆ respiratory rate (%)	∆ Heart Rate (bpm)	∆ Mean Arterial Pressure (mmHg)	∆ QRS Interval
SSCI-3	10	15	3	32.1 ± 2.0	3.8 ± 0.2	−8.5 ± 1.8	3.0 ± 0.7	12.9 ± 2.1	0.8 + 0.2
	10	30	3	43.5 ± 1.8	5.1 ± 0.2	−3.2 ± 1.8	5.0 ± 1.9	6.8 ± 8.7	0.5 + 0.2
	30	15	3	107.5 ± 4.1	12.7 ± 0.5	−6.2 ± 5.4	5.6 ± 1.7	13.8 ± 0.7	0.3 + 0.4
	30	20–21	3	171.2 ± 10.2a	20.2 ± 1.2a	−100	NA	NA	NA
SSCI-4	3	15	1	11.4	5.4	−24.7	−3.5	6.4	2.4
	3	30	1	17.6	8.4	−26.1	−5.6	2.4	4.5
	10	15	1	31.5	15.0	−17.8	6.6	−5.5	7.6
	10	25	1	39.3a	18.7a	−100	NA	NA	NA
	30	11	1	89.7a	42.7a	−100	NA	NA	NA
SSCI-5	30	15	3	38.2 ± 1.6	4.9 ± 0.2	−13.0 ± 3.4	−1.7 ± 1.3	14.0 ± 2.1	1.5 ± 1.1
	30	30	3	42.1 ± 1.2	5.4 ± 0.2	−10.2 ± 2.6	−3.7 ± 2.8	13.3 ± 5.5	4.6 + 3.2
	60	15	2	67.7	8.7	−15.6	1.0	5.2	5.2
	60	21–23	2	154a	19.7a	−100	NA	NA	NA
SSCI-1	7	15	3	54.4 ± 3.2	3.6 ± 0.2	0.8 ± 1.8	0.5 ± 2.2	4.1 ± 0.3	1.4 ± 0.6
	7	30	3	76.7 ± 4.1	5.1 ± 0.3	4.5 ± 1.7	−1.8 ± 3.7	2.7 ± 0.5	2.5 ± 1.1
	14	15	3	88.3 ± 5.6	5.9 ± 0.4	1.9 ± 1.6	−1.4 ± 1.5	2.2 ± 1.1	0.4 ± 0.6
	14	30	3	104.0 ± 5.2	7.0 ± 0.3	4.0 ± 1.0	−0.5 ± 2.9	2.3 ± 2.5	0.4 ± 0.7
	30	15	3	230 ± 12	15.4 ± 0.8	−10.1 ± 0.9	−0.4 ± 1.2	10.5 ± 1.5	3.7 ± 0.9
	30	30	3	252 ± 4	16.9 ± 0.3	−24.8 ± 3.3	2.1 ± 1.4	4.6 ± 1.9	3.9 ± 0.8
SSCI-6	1	15	3	1.4 ± 0.2	0.025 ± 0.004	−18.3 ± 9.3	−0.1 ± 0.4	8.2 ± 7.9	1.0 ± 0.8
	1	30	1	1.5	0.027	−7.8	1.7	7.5	1.7
	1	20–23	2	2.8a	0.050a	−100	NA	NA	ND

a just prior to death

**Table 3.** Inhibition of contractility in *ex vivo* HMD assay of inhibitors **SSCI-1, 3–6**.

Compound	*Ex vivo* HMD IC_50_ (μM)
SSCI-3	2.7
SSCI-4	8.9
SSCI-5	11
SSCI-1	>30
SSCI-6	130% at 10

### Parallel assays to measure system physiology

Respiratory activity is driven by action potential firing in the phrenic nerve. These action potentials then drive contraction of the diaphragm, leading to inspiration. To better understand the effects observed in the in vivo respiratory experiments, we sought additional methods for characterizing the SSCIs, including profiling in a rat hemisected diaphragm assay and a rat phrenic nerve activity assay.

#### Rat hemisected diaphragm assay

**Figure 4.**
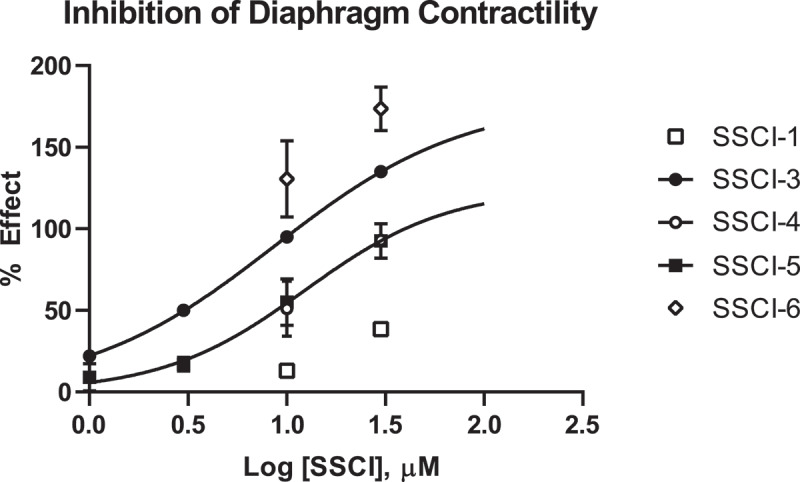
Dose Response Data for NaV1.7 Inhibitors in Rat HMD Assay.

The ex vivo hemisected diaphragm preparation (HMD) measures the contractility of the diaphragm muscle as a result from tonic activation of the phrenic nerve [24]. This preparation enabled us to determine if the compounds affected the phrenic nerve or the diaphragm muscle directly. In the HMD assay, compound effect is normalized to the amount of relaxation observed with 3 μM d-Tubocurarine (as defined in the Methods, typically 60–70% of the maximal relaxation of the muscle); this level of relaxation was independently determined for every tissue used in the study and termed the 100% inhibition level. Concentration-response data for the five SSCIs are presented in Figure 4. As shown, the compounds demonstrated a range of potencies in the rat HMD assay. Calculated IC_50_ values for each SSCI in the HMD model are reported in Table 2. Notably, an IC_50_ value for **SSCI-1** could not be determined in the HMD assay as less than 50% inhibition was observed at the highest concentration tested of 30 μM. **SSCI-6** produced supramaximal inhibition in the rat HMD assay at both 10 and 30 μM (Figure 4 and Table 3). This supramaximal inhibition precluded the determination of an IC_50_ value in the rat HMD assay.

Inhibition of rat diaphragm contractility is plotted for each SSCI as a function of compound concentration tested in the assay. The percent effect is normalized to the effect measured with 3 µM d-Tubocurarine as described in the Methods; inhibition greater than the d-Tubocurarine is then reported as greater than 100% effect. Compounds that achieved at least 50% inhibition were fit with the Hill Equation to determine an IC_50_ value for that compound. Compounds that did not achieve 50% inhibition or did not show inhibition less than 100% were not fit and data is reported in Table 3 as percent inhibition at tested concentrations.

#### Rat phrenic nerve firing assay

**Figure 5.**
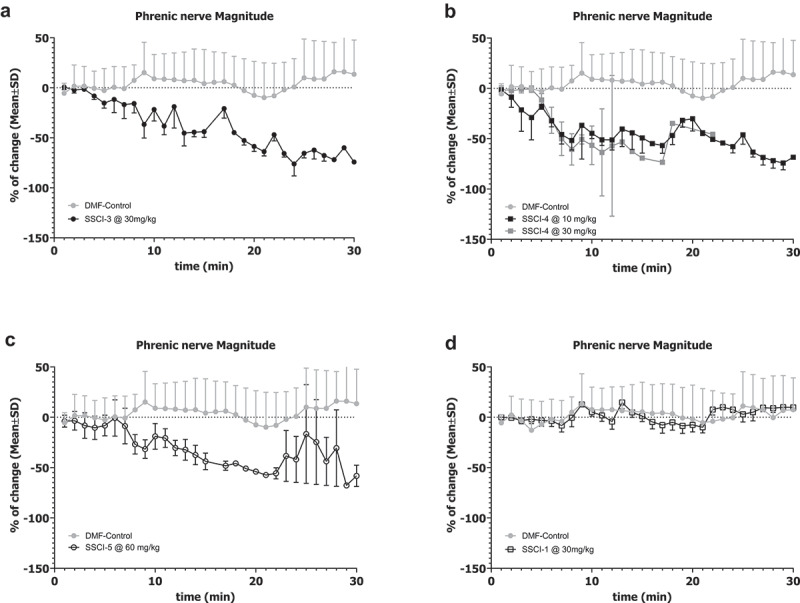
Effects of NaV1.6 Inhibitors on Rat Phrenic Nerve Activity.

The HMD assay does not distinguish between effects on the diaphragm muscle or the phrenic nerve which innervates the muscle. To measure the activity of test compounds directly on the phrenic nerve, we established a rat in vivo phrenic nerve activity assay as described in the methods. Drug concentrations were selected to result in similar unbound drug exposures as measured in the rat CV/R studies as detailed in Table 4. The effect on magnitude of phrenic nerve firing was measured at 30 minutes for **SSCI-3, SSCI-4, SSCI-5** and **SSCI-1** to allow for consistent comparison between compounds. Compounds **SSCI-3-5** consistently decreased the magnitude of the phrenic nerve electrical signal as shown in Figure 5a-c. The percent decrease in the magnitude of the signal was 74.2%, 50%, and 76.1% after administration of **SSCI-3, SS1-4**, and **SS1-5**, respectively. In contrast, **SSCI-1**, which has the lowest potency on NaV1.6 channel, did not show any decrease in the phrenic nerve signal over the course of the recordings (Figure 5d).

Changes in the magnitude of phrenic nerve electrical activity were measured for each SSCI as described in the Methods. Each panel depicts the change in average magnitude from the vehicle baseline as a function of time. The gray symbols in each panel are from vehicle (DMF) animals, while the black symbols are from compound treated animals. Error bars represent SEM and are unidirectional for clarity. Compound infusion started at time 0; a stable recording in vehicle had been obtained as a baseline prior to compound infusion and is not represented here. **SSCI-3** (A, solid black symbols), **SSCI-4** (B, closed black squares), and **SSCI-5** (C, open black circles) all demonstrated a greater than 50% depression of phrenic nerve activity magnitude. **SSCI-1** (D, open black squares) did not demonstrate any significant change in phrenic nerve activity magnitude over the course of the experiment.

**Table 4.** Plasma exposure in phrenic nerve activity assay.

Compound	Dose (mg/kg)	Time (min)	n	Total [plasma] (μM)	Unbound [plasma] (μM)
SSCI-3	30	15	3	32.115 ± 3.402	3.78 ± 0.4
	30	22–30	3	43.549 ± 3.116	5.138 ± 0.36
SSCI-4	10	15	3	55.315 ± 5.498	23.30 ± 2.61
	10	30	2	61.573	29.27
	30	10	1	91.385	43.45
	30	25–33	3	108.234 ± 9.606	51.46 ± 4.56
SSCI-5	60	15	5	82.704 ± 15.213	10.58 ± 1.94
	60	30	4	86.186 ± 42.149	11.03 ± 5.39
SSCI-1	30	30	3	236 ± 110	15.81 ± 7.37

## Discussion

Several NaV1.7 inhibitors synthesized during our program demonstrated mortality and respiratory effects that were not cardiovascular in origin. The effects on respiratory rate were found to be dose-dependent with modest depression at lower doses up to a threshold that then resulted in complete suppression of respiration. Severe respiratory depression and mortality in anesthetized animals could be prevented with mechanical ventilation suggesting a primary effect on the respiratory system, likely either by inhibition of phrenic nerve signaling to the diaphragm or failure of the diaphragm muscle to contract the diaphragm. None of the compounds profiled in the cardiovascular/respiratory model demonstrated significant inhibition of either NaV1.5 or NaV1.4 channels in in vitro patch clamp experiments, leading to the hypothesis that inhibition of another ion channel was producing the respiratory depression. Profiling of the compounds against a range of different ion channels (Supplementary Table 1) did not reveal a pattern of off-target inhibition that could explain the respiratory effects. Of the remaining peripheral voltage-gated sodium channels that were assessed, these compounds demonstrated significant inhibition on NaV1.6 with IC_50_ potencies ranging from 0.023 μM to >30 μM.

Profiling compounds in additional assays of respiratory function indicated that inhibition of NaV1.6 was the probable cause of the respiratory depression and failure. The rat HMD assay is an ex vivo measurement of respiratory function, encompassing both the phrenic nerve and the diaphragm. Three of the four compounds tested in the ex vivo and in vivo respiratory assays had very similar potencies for inhibition of NaV1.6 (**SSCI-3-5** in Table 1). **SSCI-3-5** and **SSCI-6** demonstrated significant inhibition of diaphragm contraction. The extent of inhibition in the HMD assay correlates with potency on NaV1.6 – i.e. more potent compounds demonstrate inhibition of diaphragm muscle contraction at lower concentrations. **SSCI-1**, a compound with a significant right-ward shift in NaV1.6 potency compared to **SSCI-3** and **SCCI-4**, had little effect in the HMD assay at both 10 and 30 μM bath concentrations. On the opposite end of the spectrum, **SSCI-6**, a compound with nanomolar potency on NaV1.6, demonstrated supramaximal inhibition in the HMD assay at both tested concentrations of 10 and 30 μM. **SSCI-6** is in the same structural class as **SSCI-3** and inhibits NaV1.6 channels with a calculated IC_50_ of 0.023 μM in in vitro electrophysiology experiments (Table 1 and Figure 2). Additionally, the off-target activity profile for **SSIC-6** is similar to **SSCI-3** (Supplementary Table 1).

**Figure 6.**
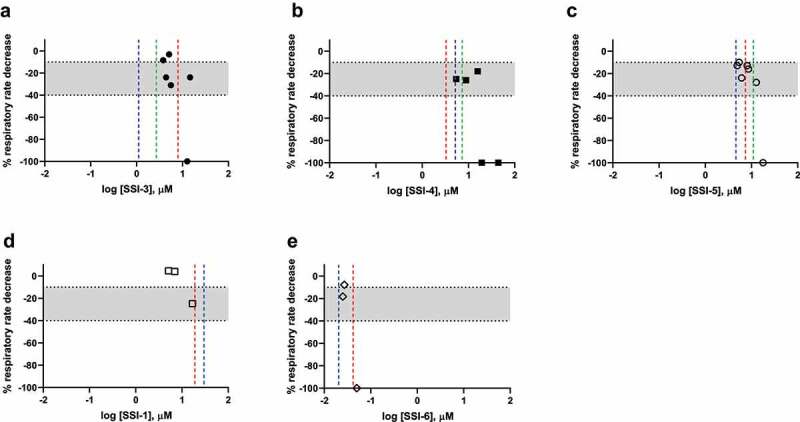
Effect of NaV1.7 Inhibitors on Rat Respiratory Rate (blue = human NaV1.6 IC_50_; red = rat NaV1.6 IC_50_, green = HMD IC_50_).

The change in respiratory rate observed in the rat CV/R assay with the measured ex vivo potency in the HMD assay and the in vitro NaV1.6 potency for each compound can be compared to better understand the relationship between these three parameters. The decrease in respiratory rate for each compound was plotted as a function of the unbound plasma concentration for each test compound in Figure 6 (A-D). The dashed lines on the graphs represent the human NaV1.6 (blue) and the rat NaV1.6 (red) in vitro calculated IC_50_ value from in vitro electrophysiology experiments. The calculated HMD IC_50_ is indicated by the green dashed line. The shaded area on each dose-response curve in Figure 6 represents measurable reduction in respiratory rate in the range of 10 to 40%. This range was defined as a significant effect in the rat respiratory assay as decreases in respiration rate below 10% were within the variability of the assay. Decreases greater than 40% were not observed without respiratory collapse and mortality occurring. The small dynamic range between a measurable effect on respiratory rate and complete cessation made quantitative pharmacological analysis and dose-response assessments difficult. The rapid transition from modest reductions in respiratory rate to complete cessation suggests that the respiratory system has a built-in tolerance for partial inhibition. However, once past a threshold level of respiratory depression, there is a steep drop in respiration and full cessation and mortality is observed. For the compounds with calculated HMD IC_50_ values, the HMD IC_50_ represents a threshold concentration for the respiratory depression – i.e. measurable respiratory depression is observed near unbound plasma concentrations equivalent to the calculated HMD IC_50._ Once unbound plasma concentrations exceeded the HMD IC_50_ complete respiratory cessation was observed in the animals. Similarly, the threshold to respiratory cessation was related to the in vitro potencies measured for both the human and rat isoforms of NaV1.6. The human and rat NaV1.6 IC_50_ values were consistent for each compound in this set indicating that the effects observed in rats may translate to humans.

Each panel represents the % respiratory rate decrease (Y-axis) in the rat CVR assay as a function of free plasma exposure (X-axis) for each SSCI. The dashed lines in each panel represent the in vitro potency for human NaV1.6 (blue), rat NaV1.6 (red), or the ex vivo potency in the rat HMD assay (green). The shaded area on each graph demarks respiratory rate decrease from −10 to −40%. **SSCI-1** (D) and **SSCI-6** (E) do not have a reported HMD IC_50_ value because the compounds either did not achieve 50% inhibition (**SSCI-1**) or all concentrations produced supramaximal inhibition (**SSCI-6**) in the assay. A % respiratory decrease of 100% also indicates animal mortality in the assay.

The involvement of voltage-gated sodium channels in respiration and phrenic nerve activity was first reported in the 1960s [25,26]. Early studies demonstrated that natural toxins could either excite or inhibit phrenic nerve activity when applied to dissected nerves in ex vivo electrophysiology experiments. Activating toxins such as batrachotoxin elicited electrical activity in the phrenic nerve, while pore blocking toxins like tetrodotoxin inhibited phrenic nerve firing. Prior to this study, the involvement of NaV1.6 in respiratory function was unexpected. First described in 1967, spontaneous mutations in the NaV1.6 gene in mice (motor endplate disease or *med* mice) did not produce respiratory effects. Although spontaneous truncation mutations in NaV1.6 give rise to motor deficits (paralysis, muscle atrophy, and failure of neuromuscular transmission), with mice dying between 21 and 24 days of age [27,28] there were not any effects on respiration reported. Another spontaneous mutation (*dmu*) was described by Repentigny et al that resulted in decreased expression of the SCN8A gene [29]. Like the *med* mice, these *dmu* mice died at weaning with a dramatic loss of hindlimb control, muscle atrophy, and labored breathing. Other spontaneous mutations that result in premature stop codons result in animals that have motor deficits, but still survive to 1.5 years of age. One human mutation has been identified, a protein truncation, with a reported phenotype of cerebellar atrophy, ataxia, and mental retardation [30]. More missense variants of NaV1.6 have been identified in humans – the majority associated with epileptic encephalopathy [31]. The motor deficits observed with both mice and human mutations support the hypothesis that NaV1.6 plays a critical role in cerebellar function. The association with epilepsy has also led to the consideration of NaV1.6 as a target for epilepsy [32]. Gene and protein expression of NaV1.6 confirm its presence in the Purkinje cells of the cerebellum, cortical areas, motor neurons, and the neurons of dorsal root ganglia. No expression has been detected in bulk tissue sequencing for the lungs. However, internal expression data from our group showed NaV1.6 protein expression in both rat and rhesus phrenic nerve (data not shown). Additionally, immunohistochemical studies by McGonigal et al demonstrate expression of NaV1.6 in the Nodes of Ranvier of the phrenic nerve innervating the triangularis sterni muscle [33]. Earlier studies localize NaV1.6 to the Nodes of Ranvier in other peripheral nerves [3]. Effects from inhibition of NaV1.6 could arise from central or peripheral mechanisms. The compounds studied here are peripherally restricted, making it unlikely that inhibition of NaV1.6 in the CNS underlies the respiratory effects.

Given that NaV1.6 is the major voltage-gated sodium channel expressed in the phrenic nerve, NaV1.6 is likely a main contributor to excitatory action potentials that can be measured in phrenic nerve recordings. Furthermore, respiratory depression by local anesthetics has been observed in animal studies [34,35] and as a result of total spinal anesthesia [36] or interscalene block in shoulder surgery [37]. Inhibition of phrenic nerve firing by TTX [26] and aconitine [38] has been demonstrated previously, leading to the conclusion that phrenic nerve firing is a direct measure of voltage gated sodium channel activity. Recently, HMD blockade from mouse preparations using the spider peptide huwentoxin-IV was also attributed to Nav1.6 inhibition [39]. The lack of a respiratory effect in both mice and humans with genetic loss-of-function mutations in the NaV1.6 gene could be due to increased expression of another NaV subtype during development or other compensatory mechanisms, which counteract the loss of NaV1.6 function.

Importantly, the potency of the compounds on NaV1.7 channels does not appear to be related to the adverse respiratory effects observed. **SSCI-1**, a compound that inhibits NaV1.7 channels with nM potency without having a large inhibitory effect on NaV1.6 at 30 μM does not exhibit respiratory effect in either the rat HMD or phrenic nerve activity assay. Similarly, **SSCI-1** does not cause effects on respiration in the rat CV/R assay at unbound exposures which inhibit primarily Nav1.7 channels. However, respiratory rate is reduced at exposures that are within two-fold of the calculated NaV1.6 IC_50_ for **SSCI-1**. Conversely, **SSCI-6** is approximately 10-fold more potent at inhibiting NaV1.6 channels and inhibits respiratory function at lower exposures.

Overall, our findings demonstrate a role for NaV1.6 channels in respiratory function. Inhibition of NaV1.6 channels leads to respiratory deficits as measured in an in vivo model monitoring cardiovascular and respiratory parameters and by force of diaphragm contraction in an ex vivo model. Furthermore, inhibition of NaV1.6 channels inhibited phrenic nerve activity – the main driver of respiration in mammals. The correlation between the potency at inhibiting NaV1.6 channels in vitro and at eliciting inhibitor effects in the different models of respiratory function build confidence that these effects can be ascribed to NaV1.6 channel inhibition rather than nonselective inhibition on sodium channels. Given the expression pattern of NaV1.6 and the effects observed on phrenic nerve activity our results demonstrate that the NaV1.6 channel is an important component of phrenic nerve activity enabling/controlling respiration. As such, measuring the effect of sodium channel inhibitors on NaV1.6 channels and monitoring for adverse effects on respiration should be a key consideration when developing sodium channel inhibitors for the treatment of pain or epilepsy.

## Supplementary Material

Supplemental Material

## Data Availability

The authors confirm that the data supporting the findings of this study are available within the article and its supplementary materials.
