# Supplementary material for: Association of respiratory failure with inhibition of NaV1.6 in the phrenic nerve
Source: Channels (Austin). 2022 Oct 14;16(1):230–43. doi: 10.1080/19336950.2022.2122309 (PMC9578445; doi:10.1080/19336950.2022.2122309)
Supplement: Supplemental Material [file KCHL_A_2122309_SM6747.docx]

Association of Respiratory Failure with Inhibition of NaV1.6 in the Phrenic Nerve

Rebecca M. Klein*, Mark E. Layton*, Hillary Regan, Christopher P. Regan, Yuxing Li, Tracey Filzen, Matt Cato, Michelle K. Clements, Jixin Wang, Raul Sanoja, Thomas J. Greshock, Anthony J. Roecker, Joseph E. Pero, Ron Kim, Christopher Burgey, Christopher T. John, Ying-Hong Wang, Neetesh Bhandari, Arie Struyk, Richard L. Kraus, Darrell A. Henze, and Andrea K. Houghton

# Supporting information

Scheme S-1. Synthesis of SSI-1.^a^

*^a^*Reagents and conditions: (a) 1-(chloromethyl)-4-methoxybenzene, LHMDS, THF, -78ºC to rt, 24 h; (b) NaBH_4_, methanol, rt, 40 min; (c) N-(2,4-dimethoxybenzyl)-5-fluoro-2-oxo-N-(1,2,4-thiadiazol-5-yl)-2,3-dihydrobenzo[d]oxazole-6-sulfonamide, DEAD, nBu_3_P, THF, rt, 1.3 h; (d) TFA, DCM, rt, 0.5 h.

Step a: tert-butyl 8-(3-(4-methoxyphenyl)propanoyl)-3,4-dihydroisoquinoline-2(1H)-carboxylate: To a solution of tert-butyl 8-acetyl-3,4-dihydroisoquinoline-2(1H)-carboxylate (1.9 g, 7.0 mmol) (Layton 2013) in THF (19 mL) at -78 ºC was added LHMDS (8.4 mL, 8.4 mmol, 1M in THF). After 30 minutes, this mixture was added via cannula to a solution of 1-(chloromethyl)-4-methoxybenzene (3.3 g, 21 mmol) in THF (4.7 mL) at 0 ºC. After 1 hour at 0 ºC, the the mixture was warmed to room temperature. The crude mixture was added to a saturated solution of ammonium chloride and extracted with EtOAc. The combined organics were dried over sodium sulfate, filtered, concentrated and purified by reverse phase chromatography (40-100% acetonitrile:water, 0.1% TFA as modifier, C18 column). The desired fractions were diluted in EtOAc, washed with a saturated solution of sodium bicarbonate, dried over sodium sulfate, filtered and concentrated to give tert-butyl 8-(3-(4-methoxyphenyl)propanoyl)-3,4-dihydroisoquinoline-2(1H)-carboxylate as an orange oil (0.41 g, 15% yield), calc’d mass 395.2, observed 396.4.

Step b: tert-butyl 8-(1-hydroxy-3-(4-methoxyphenyl)propyl)-3,4-dihydroisoquinoline-2(1H)-carboxylate: To a solution of tert-butyl 8-(3-(4-methoxyphenyl)propanoyl)-3,4-dihydroisoquinoline-2(1H)-carboxylate (0.40 g, 1.0 mmol) in methanol (5.1 mL) was added NaBH4 (0.39 g, 10 mmol) in one portion. After 40 min, the reaction mixture was slowly quenched with water and extracted with EtOAc, dried over sodium sulfate, filtered and concentrated to give tert-butyl 8-(1-hydroxy-3-(4-methoxyphenyl)propyl)-3,4-dihydroisoquinoline-2(1H)-carboxylate as a pale yellow oil (0.44 g, 100% yield), calc’d mass 397.2, observed 398.

Step c: (R or S)- tert-butyl 8-(1-(6-(N-(2,4-dimethoxybenzyl)-N-(1,2,4-thiadiazol-5-yl)sulfamoyl)-5-fluoro-2-oxobenzo[d]oxazol-3(2H)-yl)-3-(4-methoxyphenyl)propyl)-3,4-dihydroisoquinoline-2(1H)-carboxylate: To a solution of N-(2,4-dimethoxybenzyl)-5-fluoro-2-oxo-N-(1,2,4-thiadiazol-5-yl)-2,3-dihydrobenzo[d]oxazole-6-sulfonamide (0.52 g, 1.1 mmol) (Pero 2017) in THF (11 mL) was added tri-n-butylphosphine (0.55 mL, 2.2 mmol), diethyl azodicarboxylate (0.35 mL, 2.2 mmol) and tert-butyl 8-(1-hydroxy-3-(4-methoxyphenyl)propyl)-3,4-dihydroisoquinoline-2(1H)-carboxylate (0.44 g, 1.1 mmol) at room temperature. After 1.3 hours, the mixture was concentrated in vacuo and purified by reverse phase chromatography (30-100% acetonitrile:water, 0.1% TFA as modifier, C18 column) and resolved by chiral chromatography to give (R or S)- tert-butyl 8-(1-(6-(N-(2,4-dimethoxybenzyl)-N-(1,2,4-thiadiazol-5-yl)sulfamoyl)-5-fluoro-2-oxobenzo[d]oxazol-3(2H)-yl)-3-(4-methoxyphenyl)propyl)-3,4-dihydroisoquinoline-2(1H)-carboxylate as a white solid (82 mg, 9%), calc’d mass 845.3, observed 846.4

Step d: (R or S)-5-fluoro-3-(3-(4-methoxyphenyl)-1-(1,2,3,4-tetrahydroisoquinolin-8-yl)propyl)-2-oxo-N-(1,2,4-thiadiazol-5-yl)-2,3-dihydrobenzo[d]oxazole-6-sulfonamide: A solution of tert-butyl 8-(1-(6-(N-(2,4-dimethoxybenzyl)-N-(1,2,4-thiadiazol-5-yl)sulfamoyl)-5-fluoro-2-oxobenzo[d]oxazol-3(2H)-yl)-3-(4-methoxyphenyl)propyl)-3,4-dihydroisoquinoline-2(1H)-carboxylate (82 mg, 0.097 mmol) in dichloromethane (0.32 mL) and trifluoroacetic acid (0.16 mL) was stirred at room temperature. After 30 minutes, the reaction mixture was diluted in methanol (2.0 mL), filtered and purified by reverse phase chromatography (5-70% acetonitrile:water, 0.1% TFA as modifier, C18 column) to give (R or S)-5-fluoro-3-(3-(4-methoxyphenyl)-1-(1,2,3,4-tetrahydroisoquinolin-8-yl)propyl)-2-oxo-N-(1,2,4-thiadiazol-5-yl)-2,3-dihydrobenzo[d]oxazole-6-sulfonamide as a white solid (41 mg, 60%), calc’d mass 595.1, observed 596.3, ^1^H NMR (500 MHz, DMSO-*d_6_*) δ 9.05-8.88 (m, 2H), 8.43 (s, 1H), 7.81 (d, J = 7.8 Hz, 1H), 7.67 (d, J = 5.7 Hz, 1H), 7.39 (t, J = 8.9 Hz, 2H), 7.25 (d, J = 7.7 Hz, 1H), 7.08 (d, J = 8.5 Hz, 2H), 6.76 (d, J = 8.6 Hz, 2H), 5.46 (dd, J = 9.3, 5.6 Hz, 1H), 4.39 (d, J = 15.7 Hz, 1H), 4.08 (d, J = 15.7 Hz, 1H), [3.7 (s, 3H)], 3.40-3.31 (m, 1H), 3.31 – 3.20 (m, 1H), 3.09 – 3.00 (m, 1H), 3.00 – 2.91 (m, 1H), 2.86 – 2.75 (m, 1H), 2.72 – 2.62 (m, 1H), 2.61 – 2.52 (m, 1H), 2.49 – 2.39 (m, 1H).


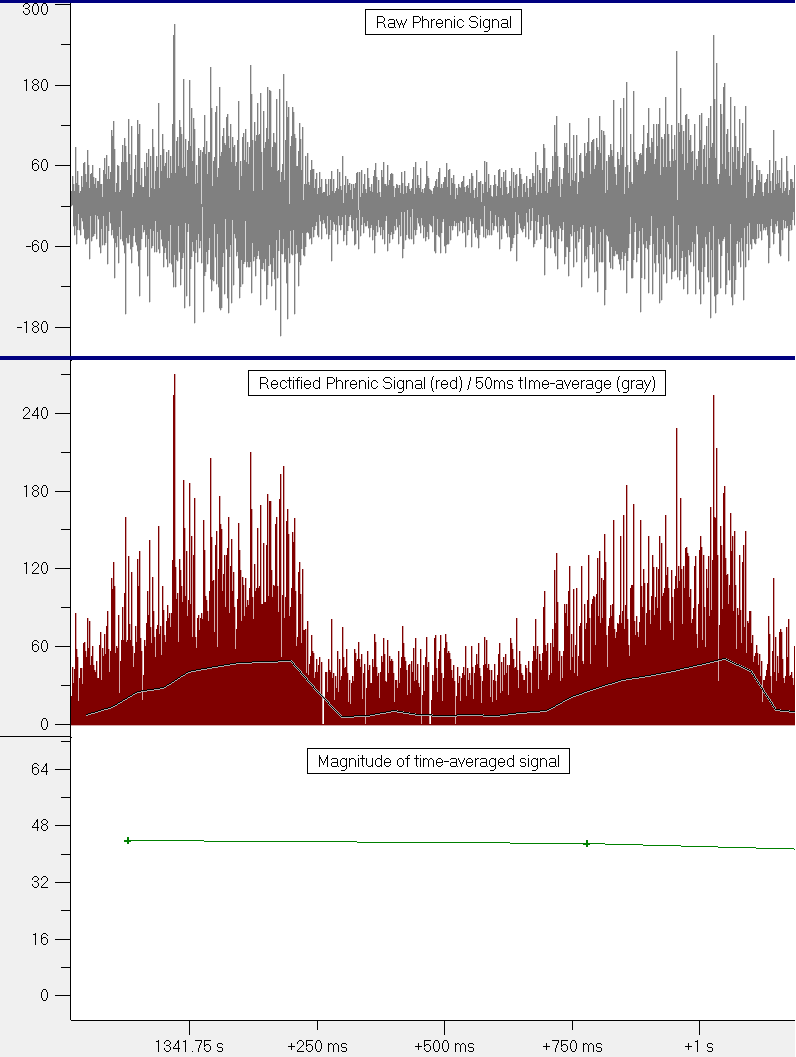


SI Figure 1 Raw Phrenic Nerve Activity and Transformed Magnitude Signal

Top panel shows the raw electrical activity recorded from a rat phrenic nerve preparation as detailed in the Methods. In the second panel, the raw signal was rectified (i.e. transformed to its absolute value, red trace) and each burst of phrenic nerve activity was defined. For each burst (complex), the baseline was subtracted (max – min) and then averaged over 50ms increments (the length of a burst) and plotted vs time (gray trace, middle panel). This resulting average was termed the magnitude and averaged over 1 minute increments to produce the trace shown in the bottom panel (green). The y-axis in all panels is the electrical signal measured in mV and the x-axis is time, measured in ms.

SI Table 1. Off target profiles of inhibitors SSI-1 to SSI-5

| **Source** | **Assay** | **SSI-5** | **SSI-3** | **SSI-4** | **SSI-1** | **SSI-6** |
| --- | --- | --- | --- | --- | --- | --- |
| In vitro | Nav1.6 bind K_i_, nM | 4.7 (n=1) | 19.8 (n=2) | 21.5 (2) | 47.1 (11) | 0.08 (n=1) |
|  | PX Nav1.6 IC_50_, nM | 946 | 3991 | 4461 | 18250 | 18 - 36 |
| Pharmacokinetics | Rat plasma protein binding | 88.20% | 52.45% | 87.20% | 93.30% | 98.20% |
| Global Counterscreen | Cav1.2 IC_50_ (uM) | >30 | >30 | 20 | >30 | 24 |
|  | MK499 K_i_ (uM) | >57 | >52 | >20.6 | >54 | 17 |
| Functional Ion Channel Panel | hERG (%inh @ 30uM) | 24% ± 4% | 16% ± 1% | 30% ± 3% | 22% ± 3% | ND |
|  | Iks (%inh @ 30uM) | -3% ± 4% | -2% ± 6% | 11% ± 2% | -1% ± 3% | ND |
| Panlabs selection | Number of assays screened | 145 | 107 | 146 | 115 | ND |
|  | Adrenergic α1, non-selective (K_i_, uM) | >10 | ND | >10 | ND | ND |
|  | Adrenergic α1D (K_i_, uM) | >10 | >10 | >10 | >10 | ND |
|  | Adrenergic α2, non-selective (K_i_, uM) | >10 | ND | >10 | ND | ND |
|  | Adrenergic α2A (K_i_, uM) | >10 | >10 | >10 | >10 | ND |
|  | Adrenergic α2B (K_i_, uM) | >10 | 1.59 | >10 | >10 | ND |
|  | Adrenergic α2C (K_i_, uM) | >10 | >10 | >10 | >10 | ND |
|  | Calcium Channel L-type (K_i_, uM) | >10 | ND | 6.21 | ND | ND |
|  | Glycine, Strychnine-sensitive(K_i_, uM) | >10 | >10 | 1.58 | >10 | ND |
|  | Histamine H1 (K_i_, uM) | >10 | >10 | 4.52 | >10 | ND |
|  | Melanocortin MC5 (K_i_, uM) | >10 | >10 | 5.02 | >10 | ND |
|  | Muscarinic, non-selective (K_i_, uM) | >10 | ND  (M1-M5 >10) | >10 | ND | ND |
|  | Nicotinic Acetylcholine (K_i_, uM) | >10 | ND | >10 | >10 | ND |
|  | NAChR α3β4 (K_i_, uM) | ND | 1.36 | ND | >10 | ND |
|  | Serotonin 5-HT2B (K_i_, uM) | >10 | >10 | >10 | >10 | ND |
|  | Transporter, Choline (K_i_, uM) | >10 | >10 | >10 | 3.19 | ND |
|  | Transporter, Dopamine DAT (K_i_, uM) | >10 | >10 | >10 | 0.98 | ND |
|  | Transporter, Norepinephrine NET (K_i_, uM) | >10 | >10 | >10 | 3.93 | ND |

ND = Not Determined

SI Table 2 – Permeability Properties of SSCIs

| Compound | BA/AB Ratio | Papp, 10e-6cm/s |
| --- | --- | --- |
| SSCI-3 | ~1.032 | 1.716 |
| SSCI-4 | ~0.9913 | 1.895 |
| SSCI-5 | <999 | 1.351 |
| SSCI-6 | ND | ND |
| SSCI-1 | ~1.096 | 1.027 |

# SI References

Layton ME, Pero JE, Fiji H, Kelly MJIII, De Leon P, Rossi, MA, Gilbert KF, Roecker AJ, Zhao Z, Mercer SP, Wolkenberg S, Mulhearn J, Zhao L, Li D (2013) Preparation of benzoxazolinone compounds with selective activity in voltage-gated sodium channels. World Intellectual Property Organization, WO2013063459 A1 2013-05-02.

Pero JE, Rossi MA, Lehman HDGF, Kelly MJIII, Mulhearn JJ, Wolkenberg SE, Cato MJ, Clements MK, Daley CJ, Filzen T, Finger EN, Gregan Y, Henze DA, Jovanovska A, Klein R, Kraus RL, Li Y, Liang A, Majercak JM, Panigel J, Urban MO, Wang J, Wang YH, Houghton AK, Layton M (2017) Benzoxazolinone aryl sulfonamides as potent, selective Nav1.7 inhibitors with in vivo efficacy in a preclinical pain model. Bioorganic & Medicinal Chemistry Letters 27(12): 2683-2688.
